# FORALL: an interactive shiny/R web portal to navigate multi-omics high-throughput data of pediatric acute lymphoblastic leukemia

**DOI:** 10.1093/bioadv/vbad143

**Published:** 2023-10-04

**Authors:** Luay Aswad, Rozbeh Jafari

**Affiliations:** Clinical Proteomics Mass Spectrometry, Department of Oncology-Pathology, Karolinska Institutet, Science for Life Laboratory, 171 65 Solna, Sweden; Clinical Proteomics Mass Spectrometry, Department of Oncology-Pathology, Karolinska Institutet, Science for Life Laboratory, 171 65 Solna, Sweden

## Abstract

**Motivation:**

Pediatric acute lymphoblastic leukemia (ALL) is the most common cancer among children worldwide. The availability of easily accessible multi-omics data provides unprecedented resources and opportunities for discovering and refining disease biology, cancer biomarkers, and drug mechanisms of action. This has led to exponential growth of omics data available in public repositories. However, delivering the useful information and knowledge extraction from this data is one of the bottlenecks of multi-omics. Presenting, navigating, and downloading ALL omics data in a user-friendly interface provide a valuable platform for biologists and clinicians to get most of the omics data. Our in-house data provides in-depth mass spectrometry-based protein abundance data for a large panel of commercially available ALL cell lines. Providing this data to the scientific community in the form of a user-friendly web-portal allows for easy and detailed exploration of the data.

**Results:**

We have developed the Functional Omics Resource of Acute Lymphoblastic Leukemia (FORALL) web-portal. FORALL is a shiny-based web portal designed to navigate in-depth mass spectrometry-based proteomics data of 51 cell lines. The proteomics data can be navigated and visualized along with matched RNA expression data as well as drug sensitivity data of 528 investigational and approved drugs.

**Availability and implementation:**

FORALL is available at https://proteomics.se/forall/.

## 1 Introduction

Acute lymphoblastic leukemia (ALL) is the most common childhood cancer. Despite a high percentage of favorable outcome cases (e.g. hyperdiploid and EV6-RUNX1 subtypes), other rare subtypes are associated with poorer prognosis (Pui *et al.* 2012). Understanding the gene expression changes at both transcriptomic and proteomics levels and their association with response to anti-cancer agents in cell lines is a key step to study clinical response and resistance to therapies in ALL. It can also help inform the development of effective combination regimens. Exploring ALL cases at the phenotype level provides an invaluable source for understanding the net outcome of genomics and transcriptomics aberrations driving ALL progression. Therefore, integrative in-depth data of protein, RNA expression was generated for 51 lymphoid lineage cell lines associated with multiple rare subtypes; 27 cell lines from the B-lineage, and 22 cell lines of T-lineage (T-ALL) in addition to 2 cell lines of Epstein-Barr virus (EBV)-transformed B-cell lines (Leo *et al.* 2022). To provide more clinical value to this dataset, drug sensitivity data for a panel of 528 approved oncological drugs as well as investigational drugs in various clinical phases was integrated in the shiny application for 43 of the cell lines. The sensitivity toward drugs was reported as selective drug sensitivity score (sDSS) (Yadav *et al.* 2014).

For straightforward exploration of this novel dataset, we introduced the functional omics resource of acute lymphoblastic leukemia (FORALL), a user-friendly shiny application designed to provide convenient access to ALL cell lines data allowing navigation, statistical analyses, and visualization in sample-wise, drug-wise, and gene-wise manners.

## 2 Methods

The mass spectrometry-based protein abundance data consists of 12 326 proteins profiled across 82 samples (51 cell lines with 31 replicates). The 12 326 proteins include 9100 proteins with full overlap across the samples. The RNA expression data consists of 54 036 RNAs profiled across 66 samples (51 cell lines with 15 replicates). The RNA and protein data share 12 292 genes profiled for RNA and proteomics data. The drug sensitivity data consists of 528 drugs profiled across 43 cell lines.

FORALL uses the Shiny R package (Chang *et al.* 2023) in R to navigate the multi-omics data of ALL. Details of materials and methods used to generate the multi-omics data was presented in Leo *et al.* (2022). FORALL is deployed on a local virtual machine using ShinyProxy (Verbeke and Michielssen 2016) which organizes and manages shiny applications instances using docker containers based on multiple users' demand. FORALL depends on multiple R packages for visualization [ggplot2 (3.4.1), plotly (4.10.1) (Sievert 2020) and visNetwork (2.1.2)] and data analyses [edgeR (3.40.2), DEqMS (1.16.0), fgsea (1.24.0), and UMAP (uwot package 0.1.14) (Robinson *et al.* 2010, McInnes and Healy 2018, Korotkevich *et al.* 2019, Zhu *et al.* 2020)]. Static and interactive plots are created via ggplot2 and plotly respectively. DEqMS is implemented for differential protein abundance and edgeR for differential RNA expression analyses. For more complex phenotypic profiling, fgsea supports gene set enrichment analysis, and UMAP is implemented for flexible high-dimensional reduction and visualization of omics data. All packages are implemented across multiple tools in the shiny app.

## 3 Results

**Figure 1.**
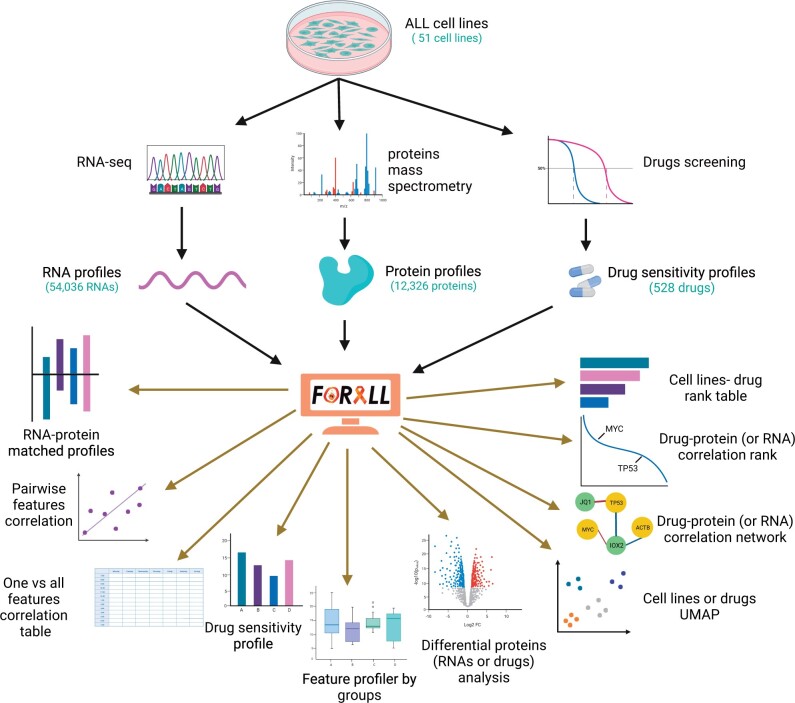
General schematic representation of FORALL modules to navigate, visualize, and hypothesis testing for ALL cell lines multi-omics data. Created in BioRender.com.

FORALL allows for the navigation, visualization, statistical, and functional analysis for multi-omics data of ALL as summarized in Fig. 1. FORALL includes multiple modules that enable:

1. visualization of protein and RNA profiles of any given gene across samples in a form of matched barplots colored based on ALL subtypes.
2. visualization of RNA–protein correlation of a given gene in B-ALL, T-ALL and all samples in a form of three interactive plotly-based scatter plots colored based on ALL subtypes.
3. visualization of RNA, protein, or drug profile across different sample groups including subtypes, cell lines lineages, consensus clusters, gender, and tissues in a form of interactive plotly-based boxplots.
4. visualization of pairwise correlation of any RNA, protein, or drug pairs profiles in B-ALL, T-ALL and all samples in a form of three interactive plotly-based scatter plots colored based on ALL subtypes.
5. visualization of pairwise correlation of any RNA, protein, or drug pairs profiles in any user-defined subset of samples in a form of an interactive plotly-based scatter plot colored based on ALL subtypes.
6. calculations of correlation table of given feature (RNA, protein, or a drug) with all other features (all proteins, all RNAs or all drugs) using all samples as well as in user-defined subset of samples.
7. visualization of gene–drug pairwise correlation in a form of a network for user-defined list of genes and drugs.
8. visualization of correlation rank of one gene versus all drugs and one drug versus all genes in a form of waterfall plot allowing the user to highlight gene(s) or drugs(s) of interest to be labeled on the plot.
9. visualization of sDSS for one drug across all cell lines or for one cell line across all drugs in a form on interactive datasheet.
10. finding differentially expressed RNAs between two user-defined groups of samples using the edgeR R package. The outcome is an interactive datasheet of standard edgeR output table.
11. finding differentially abundant proteins between two user-defined groups of samples using the DEqMS R package. The outcome is an interactive datasheet of standard DEqMS output table.
12. conducting differentially sensitivity scoring of drugs between two user-defined groups of samples using *t*-test. The outcome is an interactive datasheet with mean values of sDSS in the user-defined groups per drug along with *t*-test *P*-values and FDR adjusted *P*-values.
13. visualization of a single gene expression or drug response score in a form of two boxplots in two user-defined groups.
14. performing Uniform Manifold Approximation and Projection (UMAP) on sample-wise or drug-wise manner.
15. exploration of cell lines and drugs characteristics in user-friendly datasheets.
16. navigation of RNA-seq based fusion genes called using FusionCatcher (Daniel Nicorici *et al.* 2014) tool in user-friendly datasheet.
17. performing gene set enrichment analysis (GSEA), functional enrichment analysis for genes downloaded from differential expression or correlation analyses.

**Figure 2.**
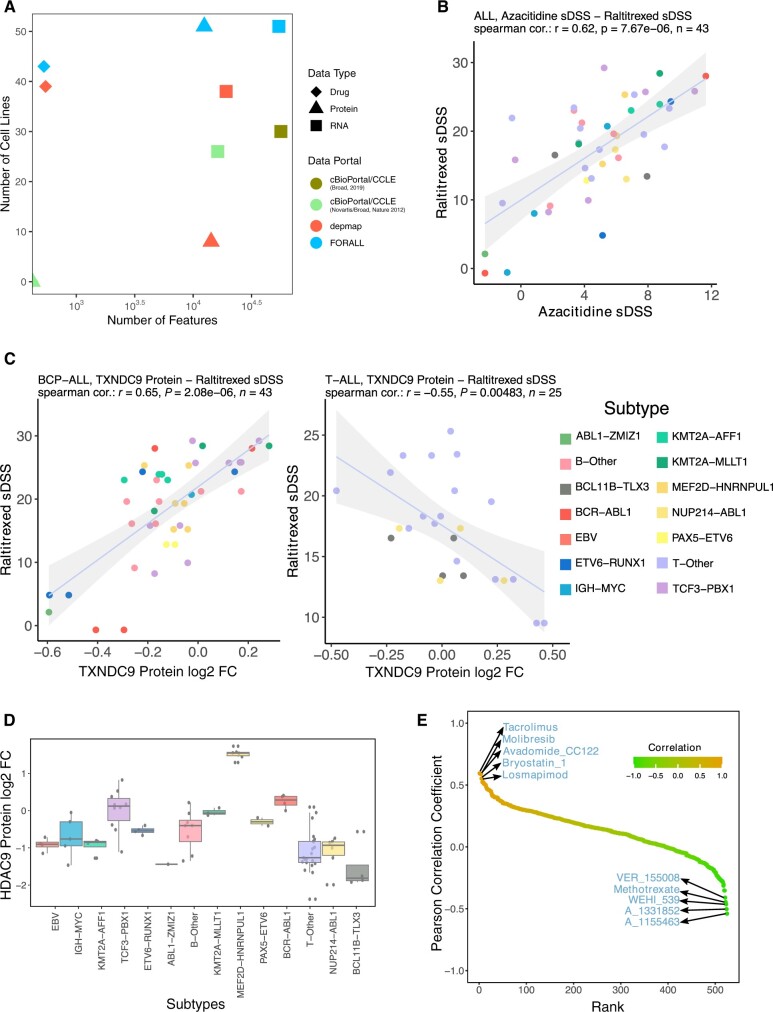
(A) Comparison of FORALL with other databases with respect to the scale of omics data related to ALL. (B) Azacitidine and raltitrexed sDSS correlation in all samples. (C) TXNDC9 protein relative abundant versus raltitrexed sDSS correlation in BCP-ALL and T-ALL lineage. (D) HDAC9 protein relative abundance across multiple ALL subtypes. (E) Ranked Pearson correlation plot between HDAC9 protein relative abundance and sDSS of all drugs in the dataset. FC, fold change; *r*, correlation coefficient; *P*, *P*-value; *n*, number of samples; sDSS, selective drug sensitivity score.

An essential feature of FORALL is allowing users to generate interactive downloadable plots and tables in html and pdf formats. Each module is accompanied with a user guide in a form of pop-up page in the corresponding module tab. We have conducted a comparison of FORALL against existing databases, focusing on the scale of omics data related to ALL, such as DepMap (Tsherniak *et al.* 2017) and cBioPortal (Cerami *et al.* 2012) (Fig. 2A). Notably, FORALL excels in several key aspects, particularly in the depth of proteomic data and the availability of ALL cell lines and their associated features. What sets FORALL apart is its exclusive focus on serving as a navigation tool tailored to this extensive collection of ALL cell lines, complete with proteomics and drug screening datasets. It's worth emphasizing that FORALL's modules are distinct from those found in other databases. They are purposefully designed for hypothesis testing and facilitating high-throughput output, setting them apart in their functionality and intended use.

There are many ways that FORALL can be utilized for hypothesis generation, testing, and validation. For example, correlation table and pairwise correlation analyses show that the nucleoside analog and hypomethylating agent, azacitidine (Müller and Florek 2010), correlated very well with raltitrexed, a TYMS inhibitor (Fig. 2B). A previous study had shown that decitabine, another nucleoside analog and hypomethylating agent, after intracellular metabolism binds to TYMS (Almqvist *et al.* 2016). Similarly in a more recent study azacitidine was also shown to bind to TYMS (Cheung *et al.* 2023). Further exploration of drug relationships can aid in finding alternative mechanisms of action of drugs in leukemic subsets.

The sub-group correlation table and pairwise correlation analyses allow users to find contrast or disagreement in correlation between two variables in cell lineage or subtype context. Most notably, we identified that abundance of the thioredoxin TXNDC9 was significantly correlated in opposing directions for numerous clinically relevant chemotherapeutics and topoisomerase inhibitors, including raltitrexed, gemcitabine, and SN-38. For BCP-ALLs, increased abundance of this protein improved sensitivity, but for T-ALLs, abundance of this protein was significantly associated with resistance (Fig. 2C). TXNDC9 has not been functionally characterized in ALL or in lymphocytes, but thioredoxins are known to carry out diverse functions in regulating both the proteasome and redox metabolism, therefore further exploration, and characterization of TXNDC9 could reveal lineage-related differences and biological dependencies and thus therapeutic implications of this protein.

In another example, differential expression analysis and cell lines groups analysis allow the identification of cell lineage or subtype specific genes or drugs. For example, HDAC9 shows high expression specifically in MEF2D-HNRNPUL1 subtype (Fig. 2D). This subtype has been shown to be sensitive to bryostatin-1 (Leo *et al.* 2022). Exploring the drugs–gene correlation rank analysis for HDAC9 confirms the high rank of overall correlation between HDAC9 and bryotsatin-1 (Fig. 2E). On the other hand, HDAC9 shows anti-correlation with the three BCL-XL inhibitors (WEHI-539, A-1155463, and A-1331852, Fig. 2E). Therefore, further exploration and characterization of the role of HDAC9 in MEF2D-HNRNPUL1 could reveal subtype-specific mechanisms associated with its involvement in MEF2D-HNRNPUL1 development and resistance to apoptotic inducer drugs.

## 4 Conclusion

FORALL is a web-based platform that allows researchers to explore multi-omics data of a large panel of ALL cell lines. FORALL provides publication-quality interactive figures from gene-centric, drug-centric and sample-centric perspectives. FORALL is designed to allow researchers without computing skills to perform hypothesis testing, visualization, and exploration of ALL data. FORALL supports the ongoing efforts for reproducible research analysis by allowing biologists to access web-based data repositories and analysis tools of novel multi-omics data.
